# Potentially clinically relevant concentrations of Cefazolin, Midazolam, Propofol, and Sufentanil in auto-transfused blood in congenital cardiac surgery

**DOI:** 10.1186/s13019-018-0747-0

**Published:** 2018-06-08

**Authors:** Gerda A. Zeilmaker-Roest, Annewil van Saet, Joost van Rosmalen, Soma Bahmany, Antony van Dijk, Enno D. Wildschut, Dick Tibboel, Ad J. J. C. Bogers

**Affiliations:** 1 Department of Cardiothoracic Surgery, Erasmus Medical Center, Rotterdam, The Netherlands; 2 Intensive Care and Department of Pediatric Surgery, Erasmus Medical Center - Sophia Children’s Hospital, Rotterdam, The Netherlands; 3 Department of Cardio-Thoracic Anaesthesiology, Erasmus Medical Center, Rotterdam, The Netherlands; 4 Department of Biostatistics, Erasmus Medical Center, Rotterdam, The Netherlands; 5 Department of Pharmacology, Erasmus Medical Center, Rotterdam, The Netherlands

**Keywords:** Cardiac surgery, Autotransfusion, Cellsaver, Paediatric, Pharmacology, Intensive care

## Abstract

**Background:**

Use of donor blood in congenital cardiac surgery increases the risk for post-operative morbidity and mortality. To reduce the need for allogenic blood transfusion a technique for peri-operative mechanical red cell salvage is applied. Blood from the operation site is collected in a reservoir, processed, passed through a lipophilic filter and returned to the patient. Influence of this cellsaver system on coagulation, fibrinolysis and inflammatory markers is known. To our knowledge no studies have been performed on the effects of autotransfusion on drug concentrations. A clinically relevant drug dose could potentially be returned to the patient through the auto-transfused blood, leading to unwanted drug reactions post-operatively. We aimed to measure drug concentrations in blood salvaged from the operation site and in the auto-transfused blood to determine if a clinically relevant drug dose is returned to the patient.

**Methods:**

The study was performed at the Department of Cardiothoracic Surgery of a tertiary university hospital. Blood samples were taken from the reservoir, after processing before the lipophilic filter, the auto-transfused blood, and the waste fluid. Samples were stored at − 80 C and drug concentration for sufentanil, propofol, midazolam and cefazolin were measured using liquid chromatography-tandem mass spectrometry (LC-MS/MS). Drug concentrations measured in the reservoir and the auto-transfused blood were compared and the relative reduction was calculated for each patient.

**Results:**

Blood samples were taken from 18 cellsaver runs in 18 patients, age 0–13 years. Drug concentrations in the reservoir were comparable to concomitant concentrations in the patient. For sufentanil 34% (median, IQR 27–50) of drug concentration was retained from the reservoir in the auto-transfused blood, for midazolam 6% (median, IQR 4–10), for cefazolin 5% (median, IQR 2–6) and for propofol 0% (median, IQR 0–0) respectively.

**Conclusion:**

Depending on the drug, up to 34% of the drug concentration salvaged from the operation site is returned to the patient through autotransfusion, potentially causing unwanted drug reactions post-operatively. Additionally, influence of a cellsaver system should be considered in pharmacological research during and after congenital cardiac surgery and could result in dose adjustments in the postoperative phase.

**Trial registration:**

Registration at the Dutch Trial Registry (NTR3579) at August 14 2012.

## Background

Allogenic donor blood is used in almost all small patients undergoing congenital cardiac surgery. This is mainly due to hemodilution caused by the use of cardiopulmonary bypass (CPB). The technique of mechanical red cell salvage is applied during congenital cardiac surgery. Equipment designed to undertake this task is routinely referred to as an autotransfusion or cellsaver system. Using the cellsaver system blood from the operation site is collected in a reservoir, processed, passed through a lipophilic filter after which it is returned to the patient. The use of donor blood during surgery increases post-operative morbidity, mainly infections [1]. The cellsaver system is used to reduce the need for allogenic blood transfusion and may therefore improve the outcome after surgery. The auto-transfused blood is usually returned to the patient postoperatively on the paediatric intensive care unit (PICU).

Influence of this cellsaver system on coagulation, fibrinolysis and inflammatory markers is known [2, 3]. In contrast, published literature on the effects of blood loss, volume replacement and use of a cellsaver system on plasma drug concentration is limited. Sue et al. investigated the effect of surgical blood loss and fluid replacement on antibiotic pharmacokinetics in adult patients during cardiac surgery and determined the total cefazolin plasma concentration at several time points [4]. In their study a cellsaver system was used in six of eight patients. Reinfusion of cefazolin-containing blood did not appear to substantially effect post-operative plasma cefazolin concentration in this study. However, cellsaver use was not specified per patient and fluid management during surgery will have changed since 1989. The authors state that major pharmacological effects may occur through infusion of auto-transfused blood in drugs that are highly protein bound or hydrophilic. Rohling et al. showed that plasma concentration of muscle relaxants were stable in autologous blood that was predonated after induction of anaesthesia and returned by the end of surgery without processing with a cellsaver system. Recurarization occurred in two out of 18 studied patients [5].

To our knowledge, drug concentration in auto-transfused blood and influence of a cellsaver system on plasma drug concentrations has not been investigated in infants and children. However, based on published literature, highly protein bound or hydrophilic drugs could persist in the auto-transfused blood. In our clinic, propofol, midazolam, sufentanil and cefazolin are routinely used anaesthetic agents that fit this risk profile. Our hypothesis was that potentially relevant drug doses of propofol, midazolam, sufentanil and cefazolin could be returned to the patient through the auto-transfused blood. This influence could be important, because it may lead to unwanted drug reactions post-operatively.

We aimed to measure drug concentrations in blood salvaged from the operation site and in the blood processed by a cellsaver system during congenital cardiac surgery to determine if a clinically relevant drug dose is returned to the patient.

## Methods

The study was performed at the Department of Cardiothoracic Surgery of a tertiary university hospital. Induction and maintenance of anaesthesia was performed by the attending anaesthesiologist as per local protocol. Either inhalation induction was performed with sevoflurane, or intravenous induction with midazolam or propofol, sufentanil and pancuronium. Maintenance of anaesthesia was performed with continuous infusions of sufentanil, midazolam or propofol. Cefazolin was administered before, during and after the surgical procedure. CPB technique was dependent on patient weight and operation procedure, and will be described extensively in the CPB-PHARM study publications. No patient underwent deep hypothermia or circulatory arrest during the procedure. We used an Electa cellsaver system (Livanova, München, Germany), with a 55 ml processing bowl. Cellsaver processing was done as per local protocol, according to manufacturer’s settings. Blood from the operation site was mixed in the suction tubes with heparinized normal saline to prevent clotting in the tubes. Washing of the cellsaver blood was done with NaCl 0.9%. After processing, the cellsaver blood was filtered with a Pall Lipiguard blood filter (Haemonetics S.A., Signy, Switzerland), resulting in the end product, the auto-transfused blood. All residual blood volume from the CPB system was also directly transferred to and processed by the cellsaver system after decannulation. The auto-transfused blood is composed according to the standard of the manufacturer, with 55–60% erythrocytes in NaCl 0.9%.

Blood samples were taken from the reservoir, after processing of salvaged blood before the lipophilic filter, the auto-transfused blood, and from the waste fluid. Samples were collected at the end of surgery. If there was a need to run the cellsaver system both during and after surgery, samples were taken after the first run for best comparison. Samples were stored at 4^o^ Celsius until processing. Samples were centrifuged (10 min at 3600 rpm) and the plasma transferred to polypropylene cryogenic vials with polypropylene screw caps (Sarstedt Aktiengesellschaft & Co, Nümbrecht, Germany). Samples were stored at -80^o^ Celsius until analysis.

Drug concentration were measured at the pharmacological laboratories of the Erasmus MC. A certified research technician from the ISO certified pharmacy laboratory performed the FDA validated drug analyses. In all analyses quality control samples are included, as is obliged in FDA analyses and ISO and GCP certified laboratory. Drug concentrations for propofol (Fresenius Kabi Nederland BV, Zeist, the Netherlands), midazolam and midazolam metabolites (Actavis Group PTC ehf., Hafnarfjördur, Iceland) were measured using LC-MS/MS (Waters Corp., Milford, MA, USA). Drug concentration for sufentanil (Hameln Pharma Plus GmbH, Hameln, Germany) and cefazolin (Kefzol®, Eurocept BV, Ankeveen, the Netherlands) were measured using LC-MS/MS (Thermo Fisher Scientific, Waltham, MA, USA).

Lower and upper limits of quantification (LLOQ and ULOQ, respectively) were: sufentanil, LLOQ 0.25 mcg/L, ULOQ 50 mcg/L; propofol LLOQ 100 mcg /L, ULOQ 25000 mcg /L; midazolam LLOQ 2 mcg/L, ULOQ 2400 mcg/L; OH-midazolam LLOQ 3 mcg/L, ULOQ 2300 mcg/L, midazolam glucuronide LLOQ 10 mcg/L, ULOQ 3000 mcg/L; cefazolin LLOQ 1 mg/L, ULOQ 100 mg/L. Drug concentrations for propofol were not measurable after auto-transfusion and were treated as zero in the analysis.

### Samples size calculation

The sample size was set at 18 patients. Due to a lack of published literature on this subject, a formal sample size calculation or power analysis was not considered feasible. The chosen sample size of 18 patients should be sufficient to estimate the median and the variability of the drug concentrations with reasonable precision.

### Statistical analysis

The ratio between drug concentrations measured in the reservoir and drug concentrations measured in the auto-transfused blood was calculated for each patient. The distributions of the drug concentrations in the reservoir and in the auto-transfused blood and their ratios were summarized with the median and the interquartile range (IQR) per drug, to give an indication of the percentage of the drug concentration remains in the auto-transfused blood. The correlation between the absolute drug concentrations measured in the reservoir and concentrations in the auto-transfused blood were calculated using Spearman’s rank correlation coefficients. Drug concentrations did not follow a normal distribution and were log(10) transformed in order to calculate the relative reduction. Relative reduction per drug was calculated by subtracting the log(10) transformed concentration in the auto-transfused blood from the log(10) transformed concentration in the reservoir. The relative reduction per drug is plotted against the reservoir concentration to predict the relative reduction per starting concentration in Fig. 3a-d, together with a linear regression line. The R^2^ was used to show the predictive value of the drug concentration in the auto-transfused blood based on the starting concentration in the reservoir, with a R^2^ of 0 representing no correlation and a R^2^ of 1 representing a perfect correlation. The reported R^2^ and the 95% confidence interval (CI) in Fig. 3a-d are based on the linear regression of the log(10) transformed relative reductions on the log(10) transformed concentrations in the reservoir.

This study was a part of the CPB-PHARM study, investigating pharmacokinetics (PK) and pharmacodynamics (PD) of routinely used drugs in neonates and children during cardiac surgery with the use of cardiopulmonary bypass (approved by IRB Erasmus MC, protocol number 2011–400, Dutch Trial Registry NTR3579). Informed consent was obtained for all study participants according to Dutch law.

## Results

Blood samples were taken from 18 cellsaver runs in 18 paediatric patients. CPB was used in all surgical procedures. Patient characteristics are shown in Table 1. As shown in Table 1, the cellsaver volume processed postoperatively is larger than the total blood loss during surgery because of addition of the residual volume of the CPB. The amount of washing fluid used is mainly dependent on the processed cellsaver volume. Not all patients received all tested drugs (see Table 2).

**Table 1:** Patient characteristics

Patient Characteristic	N (mean, minimum – maximum)
Age	2 y 6 months (2 months − 13 y 11 months)
Female /male	10 / 8
Surgical procedure	
Correction ASD type 2	6
TCPC	3
Correction TOF	3
Correction CAVSD	2
Miscellaneous	4
Total peroperative blood loss (ml/kg)	12.6 (1.6–62.7)
Cellsaver volume processed (ml)	290 (135–910)
Cellsaver product after processing (ml/kg)	12 (3.9–26)
Washing fluid used (ml)	783 (300–1200)

Kg: kilogram, ml: millilitre, y: years. Surgical abbreviations: ASD: Atrial Septal Defect, TOF: Tetralogy of Fallot, TCPC: Total Cavopulmonary Connection, CAVSD: Complete Atrioventricular Septal Defect. Miscellaneous: correction Partial Abnormal Pulmonary Venous Return (1), Chauvaud procedure (1), Mitral Valve Replacement (1), Correction Subaortic Stenosis (1)

**Table 2:** Drug concentration per sample site

Drug	Patients (n)	Concentration (median, IQR)				Ratio reservoir vs autotransfused blood in % (median, IQR)
		Reservoir	After processing, before filter	Auto-transfused blood	Waste fluid	
Sufentanil (mcg/L)	18	0.27 (0.18–0.35)	0.10 (0.09–0.12)	0.10 (0.09–0.10)	0.13 (0.10–0.16)	34 (27–50)
Midazolam (mcg/L)	18	192.69 (49.06–316.42)	18.68 (4.91–29.88)	11.87(7.16–20.16)	56.48 (17.25–75.18)	6 (4–10)
1-hydroxy-midazolam (mcg/L)	18	36.56 (9.45–62.74)	6.23 (4.39–11.52)	4.67 (3.82–5.95)	3.51 (2.72–3.84)	11 (7–29)
Midazolam glucuronide (mcg/L)	18	348.21 (236.63–592.21)	36.41 (15.83–57.13)	29.22 (21.40–41.82)	114.07 (75.33–189.71)	6 (4–8)
Cefazolin (mg/L)	18	228.44 (118.47–295.85)	10.50 (6.63–17.47)	8.70 (5.13–11.44)	59.34 (40.68–77.80)	5 (2–6)
Propofol (mg/L)	6	0.65 (0.21–1.86)	0 (0–0)*	0 (0–0)*	0.18 (0.25–0.11)	0 (0–0)

*IQR* Interquartile range, *L* Litre, *Mcg* Microgram, *mg* Milligram, *n.a*. Not applicable *Drug concentrations for propofol were not measurable after autotransfusion and were treated as zero in the analysis

Patient drug concentrations for sufentanil, cefazolin, midazolam and propofol were measured for the CPB-PHARM study (MEC2011–400). This study investigates the influence of the cardiopulmonary bypass on the PK and PD of routinely used drugs during and after congenital cardiac surgery. Patient drug samples taken from the arterial catheter during surgery were compared to drug concentrations measured in the reservoir. Drug concentrations measured in the reservoir of the cellsaver system before processing were comparable to concomitant concentrations in the patient, as was expected, and are thus clinically relevant doses. Drug levels for the different drugs and compartments are shown in Table 2. For all drugs, the decrease in drug concentration was largest after washing of the cellsaver blood. The effect of the lipophilic filter further decreased drug concentrations in all drugs except sufentanil. Considerable concentrations of all drugs were measured in the waste fluid.

**Fig. 1:**
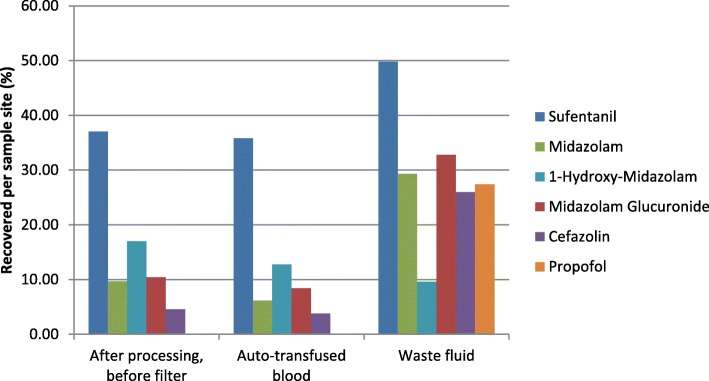
drug recovery per sample site (%). Drug concentration in the reservoir is set as 100%

Median sufentanil concentration in the reservoir was 0.27 mcg/L (IQR 0.18–0.35). Median sufentanil concentration in the auto-transfused blood was 0.10 mcg/L (IQR 0.09–0.10). Therefore 34% (median, IQR 27–50) of drug concentration was retained from the reservoir in the auto-transfused blood. Detailed concentrations per sample site and percentage recovery are shown in Table 2 and Fig. 1.

Median midazolam concentrations in the reservoir and the auto-transfused blood were 192.69 mcg/L (IQR 49.06–316.42) and 11.87 mcg/L (IQR 7.16–20.16) (6%, median IQR 4–10) respectively. Details of midazolam recovery are shown in Table 2. Biologically active midazolam metabolites, 1-hydroxy-midazolam and midazolam glucuronide, were also measured. Recovery of 1-hydroxy-midazolam and midazolam glucuronide was 11% (median, IQR 7–29) and 6% (median, IQR 4–8) respectively. Details of 1-hydroxymidazolam and midazolam glucuronide recovery are shown in Table 2 and Fig. 1.

Median cefazolin concentration in the reservoir and the auto-transfused blood was 228.44 mg/L (IQR 118.47–295.85) and 8.7 mg/L (IQR 5.13–11.44) (5%, median, IQR 2–6) respectively. Detailed concentrations per sample site and percentage recovery are shown in Table 2 and Fig. 1.

Median propofol concentration in the reservoir and in the waste fluid was 0.65 mg/L (IQR 0.21–1.86) and 0.18 mg/L (IQR 0.11–0.25) respectively. Propofol concentration could not be measured after processing and in the auto-transfused blood. Detailed concentrations per sample site and percentage recovery are shown in Table 2 and Fig. 1.

**Fig. 2:**
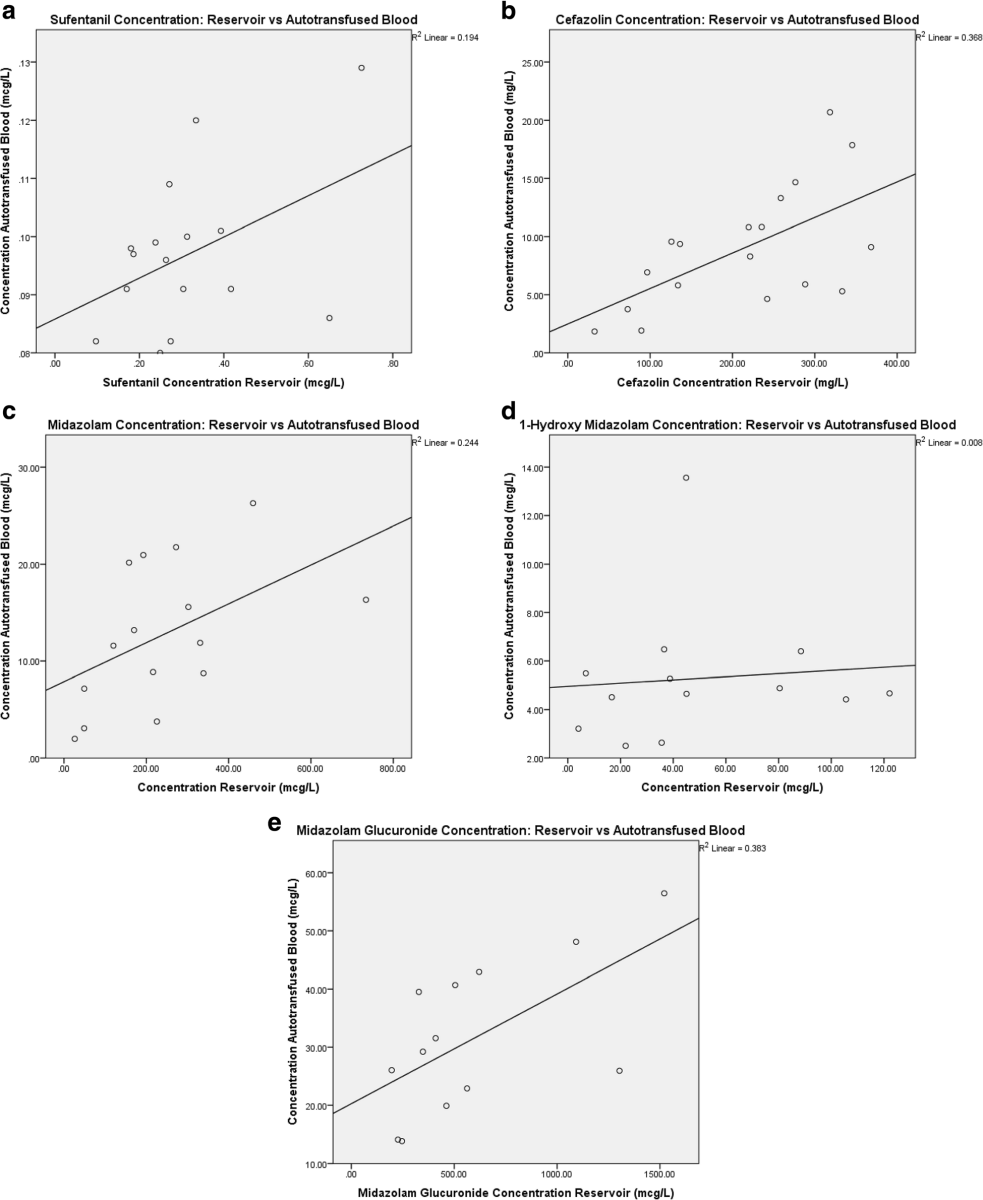
**a**-**e** correlation of drug concentration in the reservoir and the auto-transfused blood

**Fig. 3:**
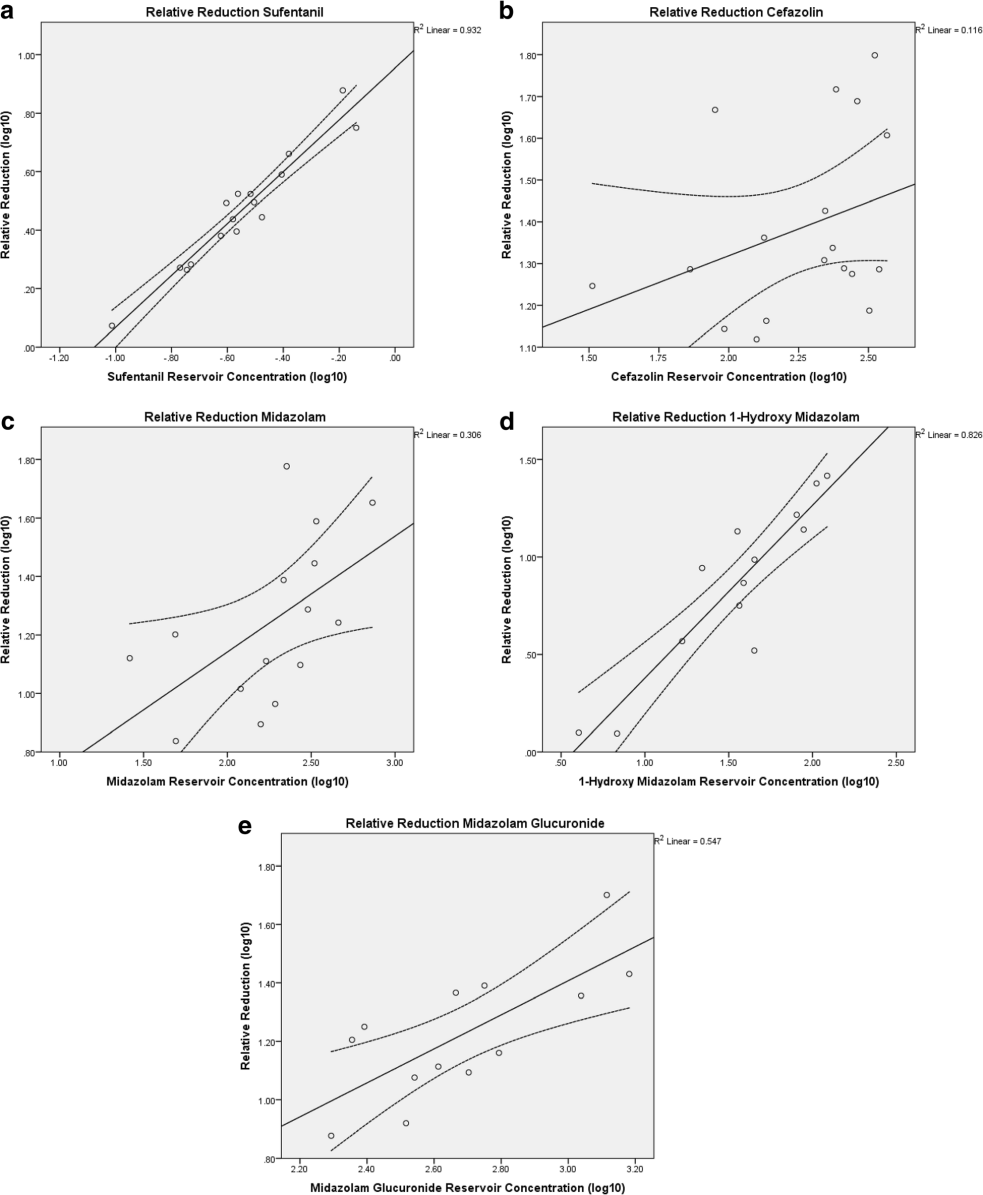
**a**-**e** relative reduction predicted by concentration in reservoir (with dotted line 95% CI)

The correlations between drug concentrations measured in the reservoir and concentrations in the auto-transfused blood are shown in Fig. 2a-e. The relative reduction per drug is plotted against the reservoir concentration to predict the relative reduction per starting concentration in Fig. 3a-e.

## Discussion

To our knowledge this is the first study to measure drug concentrations in auto-transfused blood after paediatric cardiac surgery. Drug recovery varies between drugs in the auto-transfused blood and not all drugs are found in clinically relevant concentrations. For sufentanil 34% (IQR 27–50) of drug concentration was retained from the reservoir in the auto-transfused blood, for midazolam 6% (IQR 4–10), for cefazolin 5% (IQR 2–6) and for propofol 0% (IQR 0–0), respectively. For midazolam mainly the 1-hydroxy-midazolam metabolite was recovered in the auto-transfused blood. 1-hydroxy-midazolam is at least as active as midazolam and may contribute to the overall activity of midazolam [6]. The potential influence of the return of auto-transfused blood processed in a cellsaver system on plasma drug concentration in infants and children has not previously been investigated.

Based on the literature by Sue et al. [4] and our own experience with extra corporeal membrane oxygenation (ECMO) circuit characteristics [7], the expectation was that lipophilic drugs would be sequestered in the synthetic components of the cellsaver system and the lipophilic filter and thus be prevented from returning in the auto-transfused blood whereas more hydrophilic drugs could be retained in the auto-transfused blood.

Any lipophilic compounds that are left in the auto-transfused blood after washing should be removed by the lipophilic filter. According to the manufacturer proteins and lipids should be completely washed from the end product so that it contains only erythrocytes and NaCl 0.9%. We have shown that washing of the cellsaver blood is the most effective step in clearing drugs from the auto-transfused blood. However both protein bound as well as lipophilic drugs were recovered in the end product. Interestingly sufentanil concentrations were markedly higher compared to the other lipophilic drugs. Drug characteristics of sufentanil, propofol and midazolam regarding protein binding and lipophilicity are fairly similar. With a logP of 3.95, sufentanil is highly lipophilic, with 93% protein binding, mainly to albumin. Propofol and midazolam are also both highly lipophilic with logP’s of 3.79 and 3.89 respectively, with a slightly higher protein binding than sufentanil, of 95–99 and 97% respectively. Therefore lipophilicity or protein binding do not seem to predict drug concentrations in the auto-transfused blood.

Redistribution of lipophilic drugs from erythrocytes into the auto-transfused blood could explain why lipophilic drugs are recovered. Redistribution may occur because of a shift of drugs from the erythrocyte to the NaCl 0.9% solution, or because of haemolysis of the erythrocyte. Also, measuring propofol by the precipitation method instead of LC-MS/MS may have resulted in measurable propofol concentrations after processing. Overall, the recovered absolute drug concentrations of the tested drugs where low. Therefore the absolute differences in plasma drug concentration in the patient and drug concentration in the auto-transfused blood may not be substantial.

The hydrophilic drug, cefazolin, was almost entirety washed from the auto-transfused blood. With a logP of − 0.58 cefazolin was the most water-soluble drug we have measured. Our results are probably explained by the washing of the cellsaver blood with NaCl 0.9%. It is likely that cefazolin dissolved in the NaCl 0.9% solution and was washed from the auto-transfused blood, even though cefazolin concentrations in the waste fluid were low.

Unwanted drug reactions due to auto-transfused blood may not be clinically relevant in all patients.

Most at risk for clinical effects are small patients who have had major cardiac surgery with the use of CPB, where the volume of returned cellsaver blood is relatively large compared to the patient’s own circulating volume. Also, due to long CPB time, organ perfusion will be decreased, resulting in a lower clearance of drugs from the body [8]. Returning a large volume of auto-transfused blood to a small patient with a decreased drug clearance could lead to an accumulation of drugs, resulting in adverse effects and toxicity especially for sufentanil. Also, the cellsaver blood is generally administered as a bolus, rather than a continuous infusion. This may be particularly problematic in patients who are not on mechanical ventilation when the auto-transfused blood is administered.

However, the concentration of sufentanil in the auto-transfused blood is 0.1 mcg/L. In a worst case scenario, when 1 l of auto-transfused blood is returned to a patient with a bodyweight of 3 kg, the sufentanil dose administered with the auto-transfused blood would be 0.033 mcg/kg. The target sufentanil dose at induction of anaesthesia is 0.6 mcg/kg [9]. Therefore, if the auto-transfused blood is returned to the patient during or very shortly after surgery, the sufentanil concentration in the patient is higher than the sufentanil concentration in the auto-transfused blood and dilution of plasma sufentanil concentration will occur. If the auto-transfused blood is given in a bolus sometime after sufentanil is stopped, a peak in plasma concentration may still occur.

Influence of the auto-transfused blood on adverse drug reaction is therefore dependent on weight of the child, plasma drug concentration at the time of cellsaver processing, and amount, speed and timing of administration of the auto-transfused blood.

The influence of cellsaver systems should be accounted for when performing pharmacological trials after cardiac surgery. Optimizing drug dosing in neonates, infants and children during and after cardiac surgery is important to improve clinical care, especially in an era where fast track recovery is becoming more important. Ideally, potential effects of the CPB- and cellsaver systems on drug concentrations should be incorporated in dosing advices [8]. The current study provides insight into the potential return of drugs through auto-transfused blood. Population pharmacokinetics can be used to determine subsequent dose adjustments of the investigated drugs [8, 10].

Future research should focus mainly on lipophilic anaesthetic drugs that could cause a potential adverse reaction when given to patients postoperatively through the auto-transfused blood. Also, future endeavours should aim to incorporate the results of the CPB-PHARM trial and the results of this trial into a new dosing regimen for routinely used drugs for neonates, infants and children during and after cardiac surgery. This new dosing regimen will take into account the influence of the CPB and the cellsaver system.

## Conclusion

Depending on the drug, up to 34% of the drug concentration salvaged from the operation site is returned to the patient through autotransfusion, potentially causing unwanted drug reactions post-operatively. Additionally, influence of a cellsaver system should be considered in pharmacological research during and after congenital cardiac surgery.

## Abbreviations

ASD: Atrial septal defect
CAVSD: Complete atrioventricular septal defect
CI: Confidence interval
CPB: Cardiopulmonary bypass
ECMO: Extra corporeal membrane oxygenation
IQR: Interquartile range
IRB: Institutional review board
Kg: Kilogram
L: Litre
LC-MS/MS: Liquid chromatography-tandem mass spectrometry
LLOQ: Lower limit of quantification
Mcg: Microgram
Mg: Milligram
Min: Minute
Na: Not applicable
PICU: Paediatric intensive care unit
Rpm: Rounds per minute
TCPC: Total cavopulmonary connection
TOF: Tetralogy of fallot
ULOQ: Upper limit of quantification
Y: Years
